# From the Editor’s Desk

**Published:** 2015

**Authors:** PJ Commerford

**Affiliations:** Editor in Chief

This issue provides an array of articles on a variety of cardiovascular topics ranging from clinical registries to basic laboratory science.

Most healthcare providers involved in clinical medicine and cardiology in Africa agree that rheumatic heart disease (RHD) remains common, and the consequences for affected individuals are devastating. Despite the perception of its importance, there are a limited number of detailed studies documenting with any degree of accuracy the number of patients affected. Registries serve many useful purposes, one of which describes the scale of the problem and allows planners and providers of healthcare to appropriately allocate resources and to measure whether those allocated are, over time, effective in alleviating the situation. Individual patients identified and recorded in registries may benefit from longitudinal monitoring of compliance with secondary prophylaxis, which is central to control of RHD. The data generated from good registries will greatly benefit research into RHD.

Paper-based registries are available but often not used as well as they should be. On page 227, van Dam and co-authors describe the development of an open-access mobile, compatible, electronic patient-register system based on the World Heart Federation paper-based registry forms. The system functions on mobile phones and other mobile devices, as well as on computer systems in clinics and hospitals. This is a description of an exciting development, which if implemented, could potentially change the way we measure and treat RHD.

Raynaud’s phenomenon remains one of those unusual, quirky ‘illnesses’, the pathogenesis of which we do not really understand. It causes considerable discomfort to some sufferers without serious long-term sequelae and may be effectively treated by vasodilating calcium-channel blocking agents. Karabacak and others (page 214) report that in a Turkish male cohort there was evidence of autonomic dysfunction, as measured by heart rate variability, when compared to an age- and gender-matched control cohort. The authors acknowledge that there have been other reports of autonomic dysfunction in this condition. It is not clear why the researchers chose to investigate this condition, which as they report is more common in women, in an exclusively male cohort.

Glaucoma and atrioventricular (AV) block both occur more frequently with increasing age and so it is not surprising that conduction disturbances are found in patients treated for glaucoma with β-blocker-containing eye-drops, as reported by Ozcan and co-workers (page 210). Whether the AV block is caused by the drops or not remains, as the authors state, controversial. What is important is that permanent pacing allows continuation of effective therapy for the glaucoma.

The goal of cardioprotection in the setting of acute myocardial infarction is important and has received intense attention and interest in the last decades. Despite much basic science effort and the pursuit of theoretically promising avenues, the only interventions that have proven to be clinically effective in humans have been the restoration of blood flow and maintenance of that blood flow by mechanical therapies or pharmacological treatments interfering with blood coagulation. The report by Gwanyanya and others (page 242) of the failure of magnesium to provide protection against experimentally induced myocardial infarction is another example of a theoretically impressive intervention that fails to deliver. Clinicians will continue (hopefully) to implement strategies of proven benefit and avoid untested interventions.

